# PRDX6: A protein bridging S-palmitoylation and diabetic neuropathy

**DOI:** 10.3389/fendo.2022.992875

**Published:** 2022-09-02

**Authors:** Yan Cao, Wantao Wang, Xiaorong Zhan, Yitong Zhang

**Affiliations:** ^1^ Department of Anesthesiology, Sun Yat-sen University Cancer Center, State Key Laboratory of Oncology in South China, Collaborative Innovation Center for Cancer Medicine, Guangzhou, China; ^2^ Department of Spine Surgery, The First Affiliated Hospital of Sun Yat-sen University, Guangzhou, China; ^3^ Department of Endocrinology, Southern University of Science and Technology Hospital, Shenzhen, China; ^4^ School of Life Science, Beijing Institute of Technology, Beijing, China

**Keywords:** diabetic neuropathy, DRG, S-palmitoylation, PRDX6, AE3, Cl^-^ influx, pain

## Abstract

Diabetic neuropathy is regarded as one of the most debilitating outcomes of diabetes. It can affect both the peripheral and central nervous systems, leading to pain, decreased motility, cognitive decline, and dementia. S-palmitoylation is a reversible posttranslational lipid modification, and its dysregulation has been implicated in metabolic syndrome, cancers, neurological disorders, and infections. However, the role of S-palmitoylation in diabetic neuropathy remains unclear. Here we demonstrate a potential association between activating protein palmitoylation and diabetic neuropathy. We compared the proteomic data of lumbar dorsal root ganglia (DRG) of diabetes mice and palmitoylome profiling data of the HUVEC cell line. The mapping results identified peroxiredoxin-6 (PRDX6) as a novel target in diabetic neuropathy, whose biological mechanism was associated with S-palmitoylation. Bioinformatic prediction revealed that PRDX6 had two palmitoylation sites, Cys47 and Cys91. Immunofluorescence results indicated PRDX6 translocating between the cytoplasm and cell membrane. Protein function analysis proposed that increased palmitoylation could competitively inhibit the formation of disulfide-bond between Cys47 and Cys91 and change the spatial topology of PRDX6 protein. Cl^–^HCO3^-^ anion exchanger 3 (AE3) was one of the AE family members, which was proved to express in DRG. AE3 activity evoked Cl^-^ influx in neurons which was generally associated with increased excitability and susceptibility to pain. We demonstrated that the S-palmitoylation status of Cys47 could affect the interaction between PRDX6 and the C-terminal domain of AE3, thereby regulating the activity of AE3 anion exchanger enzyme in the nervous system. The results highlight a central role for PRDX6 palmitoylation in protection against diabetic neuropathy.

## Introduction

Diabetes mellitus (DM), with an increasing prevalence, has systemic implications for health and quality of life. Among the various complications of diabetes, diabetic neuropathy (DN) is of the most importance, which can affect the peripheral nervous system, central nervous system, pain receptors, gastrointestinal systems, etc. Diabetic peripheral neuropathy (DPN), is characterized by mechanical allodynia, spontaneous pain, and paresthesia (tingling, shooting, or electric shock sensations), affecting 25 to 30% of patients with diabetes over the course of the disease (1, 2). In central nervous systems, DN can lead to decreased motility, cognitive decline, and dementia. However, currently, no treatment could clearly or effectively reverse DN in clinical. It is thus of great significance to study its molecular pathogenesis and aid in development of novel treatments for this condition.

Although the precise cellular mechanisms of DN remain poorly understood, some promising clues have emerged. Recently, various ion channels are promising topics on the mechanisms of pain inducing. The dynamic of ion channels, including sodium, potassium, calcium and chloride channels, can regulate transmission and processing of pain signals (3–5). Among those, calcium-activated chloride channels (CaCCs) intrigued us, for expressing in the neurons. Using of CaCCs current blockers can inhibit bradykinin-induced acute nociceptive pain in dorsal root ganglia (DRG) neurons (6). However, the Cl^-^HCO3^-^ ion channel, which can cause chloride influx as CaCCs, is rarely studied in diabetic neuropathic pain.

S-Palmitoylation is a dynamic and reversible posttranslational modification of palmitate onto cysteine residue of a protein, which is catalyzed by palmitoyltransferases (PATs), a family of integral membrane enzymes (7). The human PATs comprise a family of 23 zinc-Asp-His-His-Cys (ZDHHC). S-Palmitoylation regulates a variety of intracellular functions (8–10), including protein localization, protein trafficking, protein sorting, protein stability, protein-protein interaction, etc. The development of click-chemistry-based mass spectrometry technology facilitates palmitoylome profiling, revealing that palmitoylation plays a crucial role in the occurrence and development of a large number of human diseases, such as cancer, neurodegenerative diseases, inflammatory bowel disease and immune diseases (11, 12). Excitingly, emerging evidence prove that S-palmitoylation is involved in insulin functions of resistance and secretion (13). However, the mechanism of S-palmitoylation in the occurrence and development of DN is uncovered and worth exploring.

In this study, we investigated the potential role of S-palmitoylation in DN by the proteomic and palmitoylomic data, and we identified PRDX6 as a novel candidate of S-palmitoylation in diabetic lumbar dorsal root ganglia (D-DRG). Furthermore, we analyzed the gene expression of PRDX6 in human nervous system. The results also showed that S-palmitoylation modified the Cys47 of PRDX6, and this modification enhanced its interaction with anion exchanger 3 (AE3) and activated the Cl^-^/HCO3^-^ flux inducing pain in D-DRG. This study will advance our understanding of S-palmitoylation in diabetes and will provide a new nutritional approach for diabetes associated neuropathy.

## Manuscript formatting

### Identifying S-palmitoylation candidates in DRG

The D-DRG protein set was obtained from Marc’s research (14). They first established BKS-db/db mice contain a mutation of the leptin receptor, and then performed tandem mass tag labelling and mass spectrometry analysis of lumbar DRG in type 2 diabetes model. They performed functional cluster analysis of differential expression proteins and revealed 88 distinguished proteins (**Supplementary Table 1**). The data of palmitoylome in the human umbilical vein endothelial cells (HUVECs) was obtained from Wei’s research (13). They used acyl-biotin exchange chemistry technique to screen the proteins modified by S-palmitoylation in HUVECs. Proteins with a threshold of stable isotope labeling by amino acids in cell culture (SILAC) ratio of >1.5 were palmitoylation candidates. They screened out 100 S-palmitoylation candidates, and we chose these proteins for analysis in this study (**Supplementary Table 2**). We mapped and visualized those two protein-sets by Hiplot (https://hiplot.org).

### RNA-seq database

The RNA-sequencing (RNA-seq) data of the AEs family for 55 tissue types was obtained from the Consensus dataset, which was based on a combination of RNA-seq data from Internally generated Human Protein Atlas (HPA) and the Genotype-Tissue Expression (GTEx) project. The transcript per million (TPM) was normalized separately using Trimmed mean of M values (TMM) to allow for between-sample comparisons. The normalized expression (nTPM) levels were calculated for each gene in every sample.

The single cell RNA-sequencing (scRNA-seq) dataset was retrieved from the Single Cell Type Section of HPA. The data of scRNA-seq was performed on single cell suspension from tissues without pre-enrichment of cell types, and pseudo-bulk gene expression profiles were highly correlated with bulk RNA-seq profiles. The protein-coding genes were classified according to specificity into cell type enriched genes, group enriched genes and cell type enhanced genes. The cell type enriched genes were at least fourfold higher expression levels in one cell type as compared with any other analyzed cell type. The group enriched genes were enriched expression in 2-10 cell types. The cell type enhanced genes were only moderately elevated expression. These cluster results were visualized by the UMAP plot.

### The construction of PPI network

We performed the protein-protein interaction (PPI) analysis by STRING 11.5 (15). The input data was the 88 D-DRG proteins and 23 members of the ZDHHC family. We constructed full STRING network, which protein interactions included both functional and physical protein associations. The active interaction sources contained Text mining, Experiments, Databases, Co-expression, Neighborhood, Gene Fusion, and Co-occurrence. We set 0.400, indicating medium confidence, as the minimum required interaction score.

### MCL clustering

We clustered the nodes (proteins) in the PPI network by the Markov Cluster Algorithm (MCL) using the STRING tool. The MCL inflation parameter was set as 3. Edges between clusters were shown in dotted line, and each dot color was coded by its cluster.

### Enrichment analysis

We performed the Gene Ontology (GO) Functional annotation and Kyoto Encyclopedia of Genes and Genomes (KEGG) pathway enrichment for the 88 D-DRG proteins and the 23 ZDHHCs. We defined Strength as *Strength = lg (b/e)*, which was used to describe the enrichment effect. Here *b* refers to the number of proteins in the D-DRG network that were enriched in a term; *e* denotes the number of proteins that we expected to be enriched with this term in a random network of the same size from the genome. We used the False Discovery Rate *(FDR)* to describe the significance of the enrichment. FDR was the *p-values* corrected for multiple testing within each category using the Benjamini–Hochberg procedure.

### S-palmitoylation site prediction

We used the CSS-Palm software (version 4.0) to predict the S-palmitoylation site of PRDX6 and CANX based on their protein sequence in FASTA format. CSS-Palm 4.0 included a forth-generation of Group-based Prediction System (GPS) algorithm and the training data set contained 583 palmitoylation sites from 277 distinct proteins. The Particle Swarm Optimize (PSO) was also integrated to GPS. The leave- one-out validation and 4-, 6-, 8-, 10-fold cross-validations were performed to evaluate the prediction performance and system robustness.

### Protein domain analysis

We performed protein domain analysis for PRDX6 based on its protein sequence by the HMMER tool. HMMER was a tool for biosequence analysis using profile hidden Markov models. The functional domain analysis of the AE family was performed based on their protein sequence in the InterPro protein families and domains database (16).

### Immunohistochemistry

The Immunochemistry (IHC) data of PRDX6 was obtained from the HPA database. We chose the IHC results of PRDX6 stained by the antibody of CAB008663. The antibody staining in the cell types of glial cells and neuronal cells in the human brain was reported as not detected, low, medium, or high. This score was based on the staining intensity and fraction of stained cells.

### Immunofluorescence Staining

The indirect immunofluorescence (IF) microscopy was used to determine the subcellular location of PRDX6 and CANX (species-specific secondary antibodies labelled by Alexa Fluor 488, green) in human cancer cell lines. The staining of nuclei was labeled by 4’,6-diamidino-2-fenylindol (DAPI) in blue, and the endoplasmic reticulum (ER) was labeled by Alexa Fluor 647 staining for calreticulin (yellow). All IF data was obtained from the HPA database.

## Results

### PRDX6 is a palmitoylated candidate in diabetic DRG

**Figure 1:**
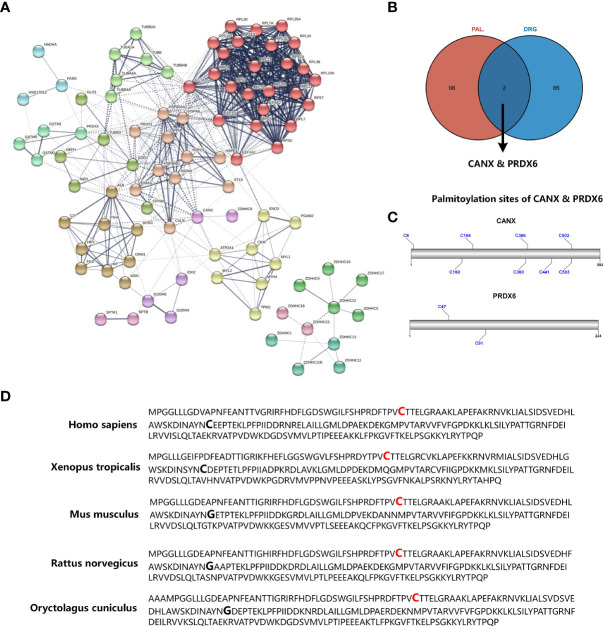
Identifying significant proteins modified by S-palmitoylation in DRG proteome of diabetic mice. **(A)** PPI network analysis of differential expression proteins in diabetic mice DRG and the ZDHHC protein family. The edges between each node indicate both functional and physical protein associations, and the line thickness indicates the strength of data support. The edges between MCL clusters were set as dotted lines. **(B)** Venn diagram showed the mapping results of DRG proteins and S-palmitoylation candidates identified by acyl-biotin exchange and mass spectrometry. **(C)** The prediction results of CANX and PRDX6 S-palmitoylation sites. **(D)** Sequence alignment among the PRDX6 amino residues of Homo sapiens, Xenopus tropicalis, mus musculus, Rattus norvegicus, and Oryctolagus cuniculus. Palmitoylation sites of Cys47 that are conserved in all species are shown in red bold type, and Cys91, not conserved in all species, are shown in bold type only. PPI, protein-protein interaction; MCL, Markov Cluster; DRG, dorsal root ganglia; Cys, cysteine.

Marc et al. have established type 2 diabetes model (BKS-db/db mice) and characterized the proteome of D-DRG by tandem mass tag labelling and mass spectrometry analysis (14). In the human body, S-palmitoylation is catalyzed by the ZDHHCs family. To explore the association between diabetic neuropathy and S-palmitoylation, we performed PPI analysis for Marc’s D-DRG proteome data and 23 ZDHHCs. Since clustering analysis of the nodes in the PPI network can further mine the potential relationships of the nodes in the network, we performed MCL clustering for the nodes of the PPI network. As shown in **Figure 1A**, the edge and cluster between CANX and ZDHHC6 was the only interaction between D-DRG proteins and ZDHHCs. Wei and colleges performed quantitative proteomic profiling of human endothelial cells using stable isotope labeling by amino acids combined with acyl-biotin exchange chemistry in cell culture and identified ≈ 380 putative palmitoylated proteins (13). To further discover D-DRG associated S-palmitoylation candidates, we mapped the palmitoylome data of HUVEC and the D-DRG proteome data. The Venn diagram showed CANX and Peroxiredoxin 6 (PRDX6) were the intersection proteins (**Figure 1B**). CANX and PRDX6 were significant proteins in D-DRG, which biological function could be regulated by the posttranslation modification of S-palmitoylation. By using the CSS-Palm software, we analyzed the FASTA data of amino sequence to predict the palmitoylation sites of CANX and PRDX6. The protein architectures revealed that CANX had eight palmitoylation sites and PRDX6 had two predicted novel palmitoylation sites of Cys47 and Cys91 (**Figure 1C**). It has been reported that ZDHHC6 was responsible for the Cys502 of CANX S-palmitoylation modification (17). However, the S-palmitoylation of PRDX6 has not been reported before. As a result, we chose PRDX6, but not CANX, for further study. Since conserved amino acid sites among species often have important biological functions, we compared the amino acid sequence of PRDX6 in five species of Homo sapiens, Xenopus tropicalis, Mus musculus, Rattus norvegicus, and Oryctolagus cuniculus. The Cys47 was conserved among species, but not Cys91 (**Figure 1D**). PRDX6 contains only one conserved cysteine residue (Cys47) rather than the two found in other six members of the PRDX family (18). These results indicated that the S-palmitoylation modification of Cys47 may play an important role in the biological function of PRDX6 in D-DRG.

### PRDX6 expression in central nervous system

**Figure 2:**
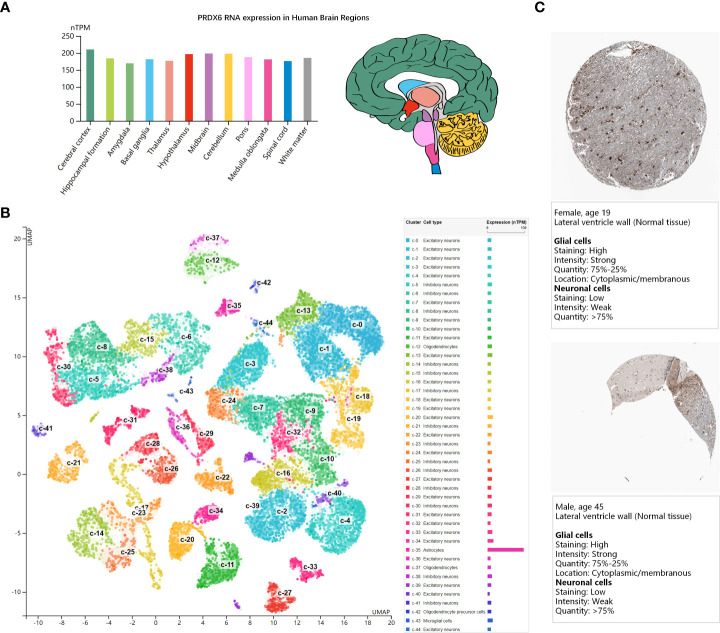
PRDX6 expression in human brain (the HPA database). **(A)** The bar-plot shows normalized RNA expression levels of PRDX6 in the 13 human brain regions. Color coding is based on brain region and the bar shows the highest expression among the subregions included. The bar-plot in red shows the distribution of PRDX6 mRNA expression in subregions of Hypothalamus. **(B)** The UMAP plot shows mRNA expression of PRDX6 in the single cell type clusters identified in brain tissue, and each dot corresponds to a cell. The bar-plot shows the mRNA expression levels of PRDX6 in each cell type cluster. The unique color assigns to each cluster. **(C)** The immunohistochemistry results of PRDX6 in histological sections from normal human brain tissues. nTPM, normalized transcript per million.

DM can affect not only the peripheral nervous system such as the DRG, but also the central nervous system. We analyzed the gene expression of PRDX6 in the brain, to explored whether PRDX6 could play a certain molecular function in the central nervous system. The bar-plot showed PRDX6 mRNA expression distributed in all the 13 regions of human brain sample (the HPA Human brain dataset), but there was low region specificity (**Figure 2A**). The UMAP plot visualized the scRNAseq data of PRDX6 in the brain cell type clusters (the HPA Human brain dataset), and PRDX6 was significantly upregulated in the astrocyte cluster (**Figure 2B**), which was similar to the expression levels of astrocyte markers of ALDH1L1, GFAP, and SLC1A3 (**Supplementary Figure 1**). Immunohistochemistry (IHC) results of human brain tissue also revealed that PRDX6 was overexpressed in the Glial cells than the neuron (**Figure 2C**), which cross proved the scRNAseq results.

### The subcellular location of PRDX6

**Figure 3:**
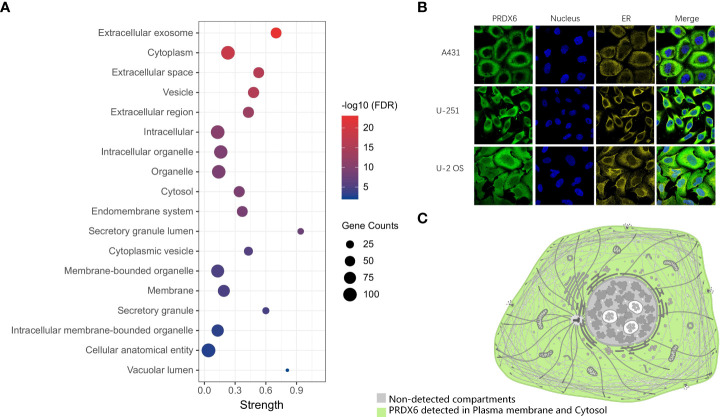
The distribution of PRDX6 in cellular components. **(A)** The GO functional categories of DRG proteins and ZDHHCs were performed by STRING. The CC terms including PRDX6 were selected and visualized in the bubble plot. **(B)** The Immunofluorescence results of PRDX6 in human cell lines indicate PRDX6 localizes in the plasma membrane and cytoplasm. **(C)** The schematic of PRDX6 subcellular localization. GO, Gene Ontology; CC, Cellular Component. A-431, Epidermoid carcinoma cell line; U-2OS, Osteosarcoma cell line; U-251 MG, Glioblastoma cell line.

We performed GO functional annotation of Cellular component (CC) for the D-DRG protein set and the ZDHHCs family (**Supplementary Table 3**). There were 18 CC terms including PRDX6, as shown in the bubble plot (**Figure 3A**). The immunofluorescence (IF) results (**Figure 3B**) indicated that PRDX6 detected in the Plasma membrane and Cytosol in the human cancer cell lines of A-431 (Epidermoid carcinoma), U-2OS (Osteosarcoma), and U-251 MG (Glioblastoma). However, CANX localized to the Endoplasmic reticulum (**Supplementary Figure 2**). The schematic showed the subcellular localization of PRDX6 to Plasma membrane and Cytosol (**Figure 3C**).

### The biological function of PRDX6

**Figure 4:**
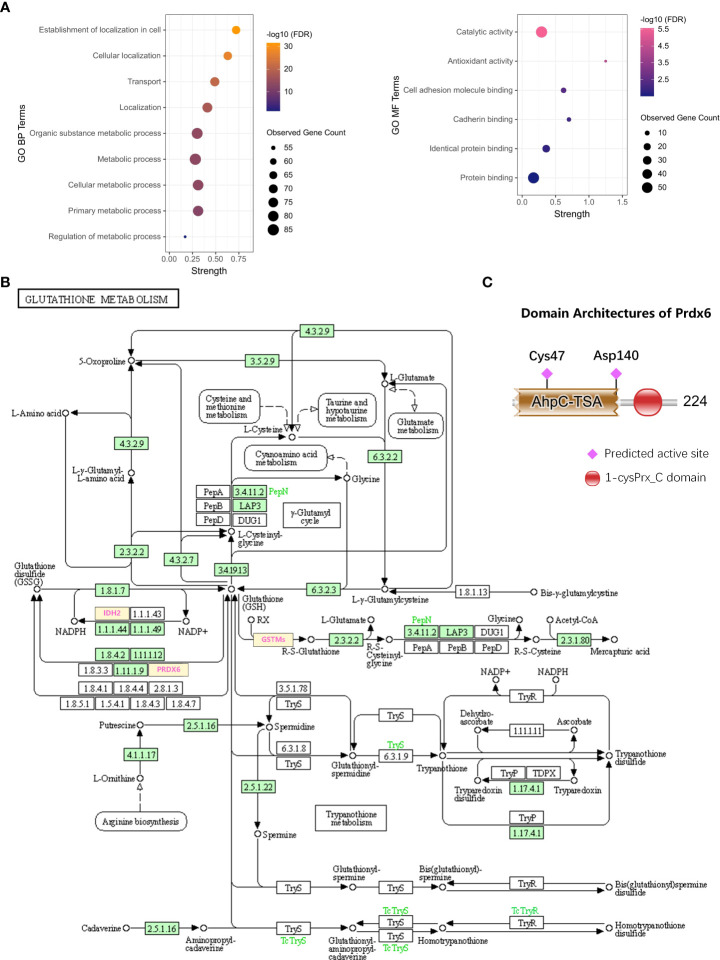
The biological function of PRDX6. **(A)** The bubble plots show the BP and MF terms of GO functional enrichment including PRDX6. **(B)** The DRG proteins of GSTM2, GSTM5, GSTM1, IDH2, and PRDX6 participate in the KEGG pathway of Glutathione metabolism (hsa00480). **(C)** The domain analysis and visualization were performed by the HMMER database. The architecture of PRDX6 protein shows the two domains of AhpC-TSA and 1-cysPrx_C, and there are two active sites of Cys47 and Asp140 in AhpC-TSA. BP, Biological Process; MF, Molecular Function; KEGG, Kyoto Encyclopedia of Genes and Genomes. Asp, aspartic.

To discover the biological function of PRDX6, the GO functional enrichment of Biological Process (BP) and Molecular Function (MF) was also performed for the D-DRG proteins and the ZDHHC family (**Figure 4A**). BP results revealed that PRDX6 was involved in 25 biological processes (**Supplementary Table 4**). It was reported that PRDX6 translocation to the plasma membrane can increase its PLA2 activity (19), and PRDX6 was one of the D-DRG protein. Thus, we focused on BP terms of Establishment of localization in cell, Cellular localization, Transport, Localization, Organic substance metabolic process, Metabolic process, Cellular metabolic process, Primary metabolic process, and Regulation of metabolic process, which associated with localization and metabolic were identified and visualized in the bubble plot. PRDX6 was involved in six MF terms, including Catalytic activity, Antioxidant activity, Cell adhesion molecule binding, Cadherin binding, Identical protein binding, and Protein binding (**Supplementary Table 5**). We also performed KEGG pathway enrichment analysis (**Supplementary Table 6**), and found PRDX6 was only enriched in the Glutathione Metabolism Pathway (**Figure 4B**). To validate the molecular function of PRDX6, we analyzed the sequence of PRDX6 by the HMMER webserver which was based on hidden Markov models. As shown in **Figure 4C**, PRDX6 had two domains of AhpC-TSA (Alkyl hydroperoxide reductase subunit C/Thiol specific antioxidant) and 1-cysPrx_C (C-terminal domain of 1-Cys peroxiredoxin). The AhpC-TSA domain had two active sites of Cys47 and Asp140, including the conserved S-palmitoylation site of Cys47, indicating the S-palmitoylation modification of Cys47 may involve in the antioxidant function of PRDX6.

### PRDX6 interacts with AE3 in nervous system

**Figure 5:**
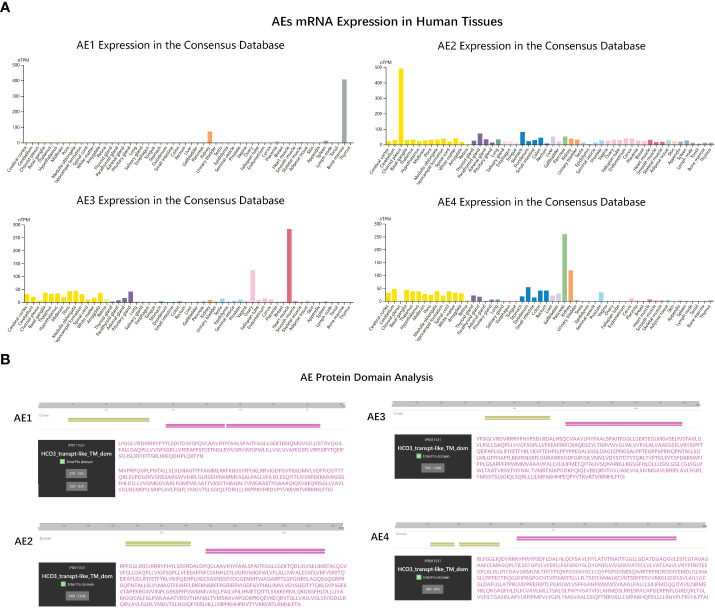
The gene expression and molecular function analysis of AEs. **(A)** The bar-plots showed tissue specificity of AEs mRNA expression in human based on RNA-sequencing data from the consensus dataset. **(B)** The protein domain analysis of the AE family (AE1-4). The amino acid sequences of key domains are shown in purple. HCO3^-^transpt-like_TM_dom, Bicarbonate transporter-like transmembrane domain.

**Figure 6:**
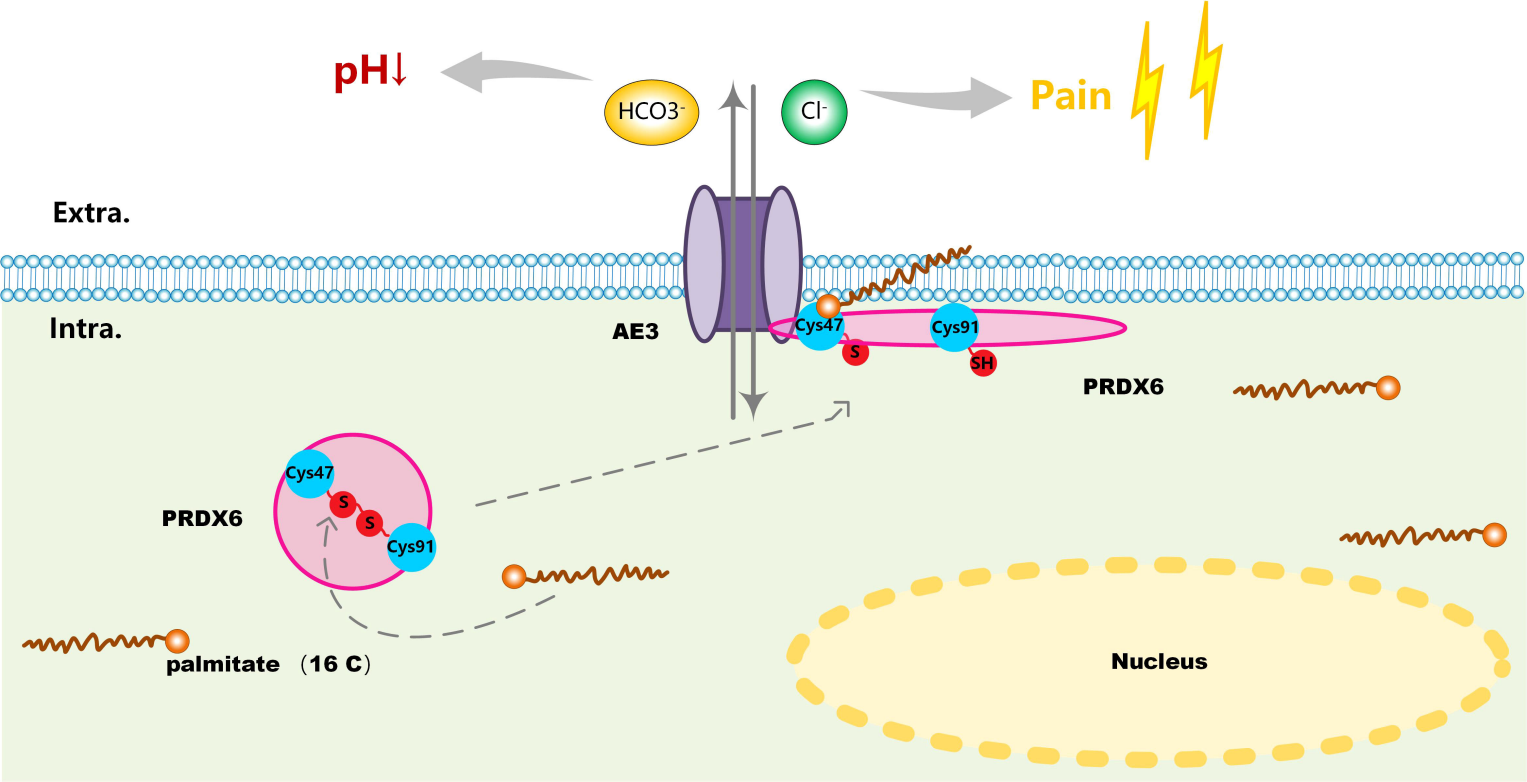
A schematic of the involved molecular mechanism that S-palmitoylation mediated reduction of disulfide bond (Cys47-Cys91) on PRDX6 activated its interaction with AE3 and induced its translocation from the cytoplasm to the plasma membrane. PRDX6-AE3 interaction enhanced the Cl^-^ influx and HCO3^-^ extra flux, causing pain and decline of pH in D-DRG.

Previously, Sara et al. demonstrated that PRDX6 is a binding partner of the C-terminal tail of anion exchanger 1 (AE1), a Cl^-^/HCO3^-^exchanger, and conformed that PRDX6-AE1 interaction could be disrupted by the Cys47Ala (alanine) mutation of PRDX6 (18). However, AE1 only expressed in erythrocytes (eAE1) and at the basolateral surface of intercalated cells in the kidney (kAE1), not detected in the nervous system. Since the AE (gene symbol Slc4) family of HCO3^-^ transporters consists of four members, we analyzed the expression of the four AEs in human tissues by RNA-seq data to identify the significant AEs in the nervous system. As shown in the bar-plots (**Figure 5A**), AE2, AE3 and AE4 were detected in the brain. Then we analyzed the functional domain of AEs to explore the protein structure in the C-terminal of them each. **Figure 5B** revealed that all of the AEs had a HCO3^-^transport domain in the C-terminal. Although the protein sequence of HCO3^-^transpont-like transmembrane domain of AE1 has a gap of 4 amino acids (aa 555-558), the four AEs have the same domain in the C-terminal. However, only AE3 has been proved to present in DRG; AE3 expression enhanced during short and long-lasting formalin-induced nociception (19), and spared nerve injury enhanced base-line AE3 expression in L4 and L5 DRGs (20). Since PRDX6 overexpressed in the D-DRG and could be modified by S-palmitoylation, we demonstrated that the palmitoylation of PRDX6 participated in the interaction between PRDX6 and the C-terminal tail of AE3 in DRG, which could activate the influx of Cl^-^ and extra-flux of HCO3^-^, resulting in pain and decrease of pH (**Figure 6**).

## Discussion

As a diabetic complication with high incidence, DN has brought a huge impact on the prognosis and life quality of individual. Since pain is the most common DN symptom, the mechanism of ion channels has attracted more attention of scholars. At present, researches on the mechanism of pain caused by DN mainly focuses on sodium and potassium channels (21–23). Recently, the role of chloride ion channels in pain has gradually attracted people’s attention. Specific chloride flux participates in signal transduction and amplification at the peripheral nerve terminal, conduce to excitability and action potential generation of sensory neurons, or crucially shape synaptic transmission in the spinal dorsal horn. Besides, inflammatory mediators can modify the chloride channels through protein-protein interaction and signaling cascades, affecting them directly. Since chloride fluxes, modulated by chloride channels, can regulate pain disorders and contribute to nociceptor excitation and sensitization, their role in nociceptive primary afferents is critical. DRG neurons express several types of chloride channel belonging to different channel families, including ligand-gated, GABA, Ca2^+^-activated chloride channels of the anoctamin (also known as TMEM16), CLC chloride channels and transporters, CFTR (cystic fibrosis transmembrane conductance regulator), Best1 (bestrophin) family, as well as VRACs (volume-regulated anion channels). Besides, Na^+^-independent Cl^–^HCO3^–^anion exchanger, which is proved for the intracellular Cl^–^ accumulation, is expressed in 60% of peptidergic and 30% of non-peptidergic DRG neurons (20). However, the Cl^–^HCO3^–^ion channel is rarely studied in diabetic neuropathic pain.

AEs, members of solute carrier families 4 (SLC4) family, including AE1, AE2, AE3, and AE4, are transporters exchanging one intracellular HCO3^‐^ for one extracellular Cl^‐^. In our results, we found AE1 was not detected in the nervous system and AE2 together with AE4 were widely expressed in various tissues with no tissue specificity. AE3 mainly distributed in the brain, heart and ovary. There were evidences that AE3 was present in DRG and participates in the development and maintenance of short and long-lasting formalin-induced nociception (19). Furthermore, it also contributed to mechanical allodynia and thermal hyperalgesia in neuropathic rats (20). Therefore, we speculate that AE3 is also related to the occurrence of diabetic neuropathy pain, and its mechanism is more worthy of our study. Previous research had shown that PRDX6 and AE1 were co-localized in human kidney by immunostaining. PRDX6-AE1 interaction at cysteine residue (Cys47) conduced to preserve AE1 function during cellular stress such as during metabolic acidosis. In this research, we analyzed the functional domain of AEs to explore the protein structure and found the four AEs had the same domain in the C-terminal. Our results proposed that the S-palmitoylation of Cys47 was associated with PRDX6-AE3 interaction and activated the influx of chloride ions caused by AE3, which could promote the occurrence of pain in DN.

The PRDX family, with peroxidase and antioxidant activity, was comprised of six members. Among those, PRDX6 was distributed in tissues of the brain, heart, kidney, lung and testis. Recently, studies had proved that PRDX6 was involved in cancer (24), inflammatory diseases (25), ischemic stroke (26), traumatic brain injury (27) and neural degenerative diseases (28). Also, PRDX6 was unique for its containing only one conserved cysteine residue (Cys47) rather than the two found in other PRDXs (29) and Cys47 was evolutionally conserved in PRDX6 among most species. Furthermore, we predicted Cys47 and Cys91 were S-palmitoylation sites of PRDX6, but only Cys47 was the active site of functional domain in PRDX6. The results of our analysis showed that PRDX6 could bind with AE3 in the DRG, as the physical interaction of PRDX6-AE1 had been proved in human kidney. However, it is not yet known the underlying mechanism of PRDX6-AE3 interaction.

We demonstrated that the S-palmitoylation of Cys47 and Cys91 could affect the steric structure of PRDX6 and promote the PRDX6-AE3 interaction. On the one hand, the Cys47 and Cys91 sites of PRDX6 could form a disulfide bond, and mutation of Cys91 alone suppressed the formation of disulfide bond without affecting the function of the Cys47 site for PRDX6 (30). As S-palmitoylation on the Cys site could reduce disulfide bond formation (31), the S-palmitoylation of Cys47 and Cys91 could change the spatial topology of PRDX6 and expose its functional domain. On the other hand, S-palmitoylation could stabilize the location of a protein on cellular membrane. As AE3 was a transmembrane ion channel, the S-palmitoylation of PRDX6 could facilitate the colocalization of AE3 and PRDX6 on the cellular membrane. Taken together, S-palmitoylation of PRDX6 at Cys47 influences its interaction with AE3 and promotes chloride influx, which is a new mechanism of DN pain.

Previous researches had shown that PRDX6 had multiple functions including glutathione peroxidase (GPx) activity, lysophosphatidylcholine acyl transferase activity and phospholipaseA2 (PLA2) activity (32). The current research mainly focused on the PLA2 activity of PRDX6. NADPH oxidase (Nox) was an important activator of inflammatory signaling pathway, which was widely reported as one of the mechanisms of diabetes. Studies had confirmed that the PLA2 activity of Prdx6 was related to Nox activation (33). The PLA2 activity inhibitor MJ33 also inhibited the activity of Nox1, suggesting that Nox1 function was related to the PLA2 activity of Prdx6 (34). Experiments had confirmed that Prdx6 overexpression could lead to inactivation of p38, MAPK and JNK signaling pathways (35). Some scholars confirmed through *in vitro* and *in vivo* studies that the PLA2 activity of Prdx6 was related to the secretion of neuroinflammatory factors IL-1β, IL-17 and IL-23, and could upregulate the expression of TLR2/4 and induce the activation of NF-κB (36). Current studies uncovered that the activity regulation of Prdx6 was affected by factors such as subcellular localization, substrate binding and post-translational modification. As one of the post-translational modifications, the S-palmitoylation of PRDX6 stabilizes its localization on the membrane, which enhanced its PLA2 activity and promoted the release of inflammatory factors (37). However, no direct effect of PRDX6 with onion channel function inducing pain had been reported. Those functions of PRDX6 corresponded to the enrichment analysis results of D-DRG proteins, which only showed that PRDX6 was related to oxidative stress, but not involved in ion channels.

At present, the research on the pathogenesis of diabetes and S-palmitoylation mainly focused on the bidirectional regulation of S-palmitoylation on pancreatic islet function. Under normal physiological conditions, specific G-proteins of H-Ras, Rac1 and Cdc42 were critical S-palmitoylation substrates for islet function and insulin secretion (38). Additionally, those G-proteins were also demonstrated to be S-palmitoylation modified under pathophysiological conditions of glucolipotoxicity, the generation of nitrosative and oxidative stress, and cytokine exposure in the islet-cell (39–42). However, little was known about the mechanism of palmitoylation and DN pain.

In our research, we compared the proteomic data of lumbar DRG of diabetes mice and palmitoylome profiling data of the HUVEC cell lines. Since palmitoylation was a non-specific reaction and thousands of proteins had been proved which could be modified by S-palmitoylation (43), so we convinced that the proteins undergo S-palmitoylation could in other cells types in addition to the HUVEC cells. However, ZDHHCs and palmitic acid were sufficient and necessary conditions for S-palmitoylation of substrates. It was not excluded that other S-palmitoylation substrates in DPN model were not expressed and/or detected in HUVEC cells. The above conclusions did not affect the hypothesis that S-palmitoylation plays an important role in the pathogenesis of DPN. Thus, this study provided a theoretical basis for further biological verification.

In this study, the protein interactions included both functional and physical protein associations, which was based on different kinds of data sources. The interaction score was based on the detailed information of these sources, which would affect the result of each PPI network generated by the STRING tool. The links only indicated the connections between each dot with medium confidence, as we set 4 as the minimum required interaction score. Most of the S-palmitoylations in the human body were catalyzed by PATs of the ZDHHC family. So, potential but weaker connections of ZDHHCs could also exist, in addition to the links visualized in this network. The PPI networks revealed potential interactions among proteins based on prior knowledge, which could potentially explain that ZDHHC6 was not even connected to the cluster of their own gene families.

Palmitoylation substrates were generally paired with PATs, and this correspondence was generally associated with both tissue specificity and subcellular colocalization. To search for the key PATs corresponding to PRDX6 in the nervous system, we analyzed the subcellular localization and tissue specificity of 23 human ZDHHCs. Immunofluorescence results demonstrated that PRDX6 was mainly localized in the cytoplasm and cell membrane, so we focused on the ZDHHCs localized in the cell membrane or cytoplasm and detected in the nervous system at the translation level (**Table 1**). To further mine ZDHHCs correlated with PRDX6, we performed the Pearson’s correlation analysis for PRDX6 and ZDHHCs by the proteomic data of human normal and cancer tissues (**Supplementary Table 7**). However, no distinguished PRDX6-ZDHHC connection emerged. Therefore, our analysis could not confirm the specific ZDHHC that catalyzes PRDX6 in D-DRG, and could only initially screen ZDHHC2, ZDHHC5, ZDHHC8, ZDHHC9, ZDHHC15, ZDHHC20, ZDHHC21 and ZDHHC24 as candidates for catalyzing the palmitoylation of PRDX6.

**Table 1:** The subcellular location and nervous system expression of ZDHHCs. (The HPA database).

	Subcellular location	Expression in nervous system
ZDHHC1	Cytosol	NA’
ZDHHC2	Plasma membrane	√
ZDHHC3	Golgi apparatus	√
ZDHHC4	NA’	√
ZDHHC5	Plasma membrane, Nucleoplasm	√
ZDHHC6	NA’	√
ZDHHC7	Golgi apparatus	√
ZDHHC8	Cytosol, Nucleoplasm	√
ZDHHC9	Endoplasmic reticulum, Golgi apparatus, Cytosol	√
ZDHHC11	Mitochondria	NA’
ZDHHC12	Nucleoplasm, Intermediate filaments	√
ZDHHC13	Vesicles, Golgi apparatus	√
ZDHHC14	Vesicles	√
ZDHHC15	Nuclear speckles, Cytosol	√
ZDHHC16	Nucleoplasm, Nuclear membrane, Cytosol	NA’
ZDHHC17	Golgi apparatus, Vesicles	√
ZDHHC18	Microtubules	√
ZDHHC19	NA’	NA’
ZDHHC20	Vesicles, Plasma membrane	√
ZDHHC21	Golgi apparatus, Cytosol	√
ZDHHC22	Plasma membrane	NA’
ZDHHC23	Nucleoplasm	NA’
ZDHHC24	Vesicles, Cytosol	√

NA’, Not available.√, detected in the human brain tissue by immunochemistry data of the HPA database.

## Conclusion

The current work identifies a post-translation regulatory mechanism for DN, PRDX6 breaks the disulfide bond (Cys47-Cys91) and translocates to the cellular membrane, which depends upon the S-palmitoylation of PRDX6 at Cys47. This mechanism causes stable PRDX6-AE3 interactions, and is responsible for the enhanced the Cl^–^/HCO3^–^ currents through AE3. This advanced our knowledge of the association between S-palmitoylation and DN. As such, these outcomes may offer novel insights for alleviating pain in DN.

## Data availability statement

Publicly available datasets were analyzed in this study. This data can be found here: https://www.proteinatlas.org/.

## Author contributions

YZ: conception and design. YC: survey and collection of data. WW: administrative support. XZ: data analysis and interpretation. All authors manuscript writing and final approval of manuscript.

## Conflict of interest

The authors declare that the research was conducted in the absence of any commercial or financial relationships that could be construed as a potential conflict of interest.

## Publisher’s note

All claims expressed in this article are solely those of the authors and do not necessarily represent those of their affiliated organizations, or those of the publisher, the editors and the reviewers. Any product that may be evaluated in this article, or claim that may be made by its manufacturer, is not guaranteed or endorsed by the publisher.
