# Supplementary material for: PRDX6: A protein bridging S-palmitoylation and diabetic neuropathy
Source: Front Endocrinol (Lausanne). 2022 Sep 2;13:992875. doi: 10.3389/fendo.2022.992875 (PMC9478578; doi:10.3389/fendo.2022.992875)
Supplement: Supplementary file 1 [file DataSheet_1.pdf]

Supplementary Material

1 Supplementary Tables

Supplementary Table 1. The list of D-DRG proteins for identifying the hub proteins in this study. (provided in a separate Excel file)

Supplementary Table 2. The protein list of S-palmitoylation for identifying the hub proteins in this study. (provided in a separate Excel file)

Supplementary Table 3. GO functional enrichment (Cellular component) results of D-DRG proteins and the ZDHHC family. (provided in a separate Excel file)

Supplementary Table 4. GO functional enrichment (Biological process) results of D-DRG proteins and the ZDHHC family. (provided in a separate Excel file)

Supplementary Table 5. GO functional enrichment (Molecular function) results of D-DRG proteins and the ZDHHC family. (provided in a separate Excel file)

Supplementary Table 6. KEGG pathway enrichment results of D-DRG proteins and the ZDHHC family. (provided in a separate Excel file)

Supplementary Table 7. The Pearson's correlation analysis results of PRDX6 and ZDHHCs protein expression level in human tissues. The proteome data was obtained from the CPTAC database. (provided in a separate Excel file)

2 Supplementary Figures

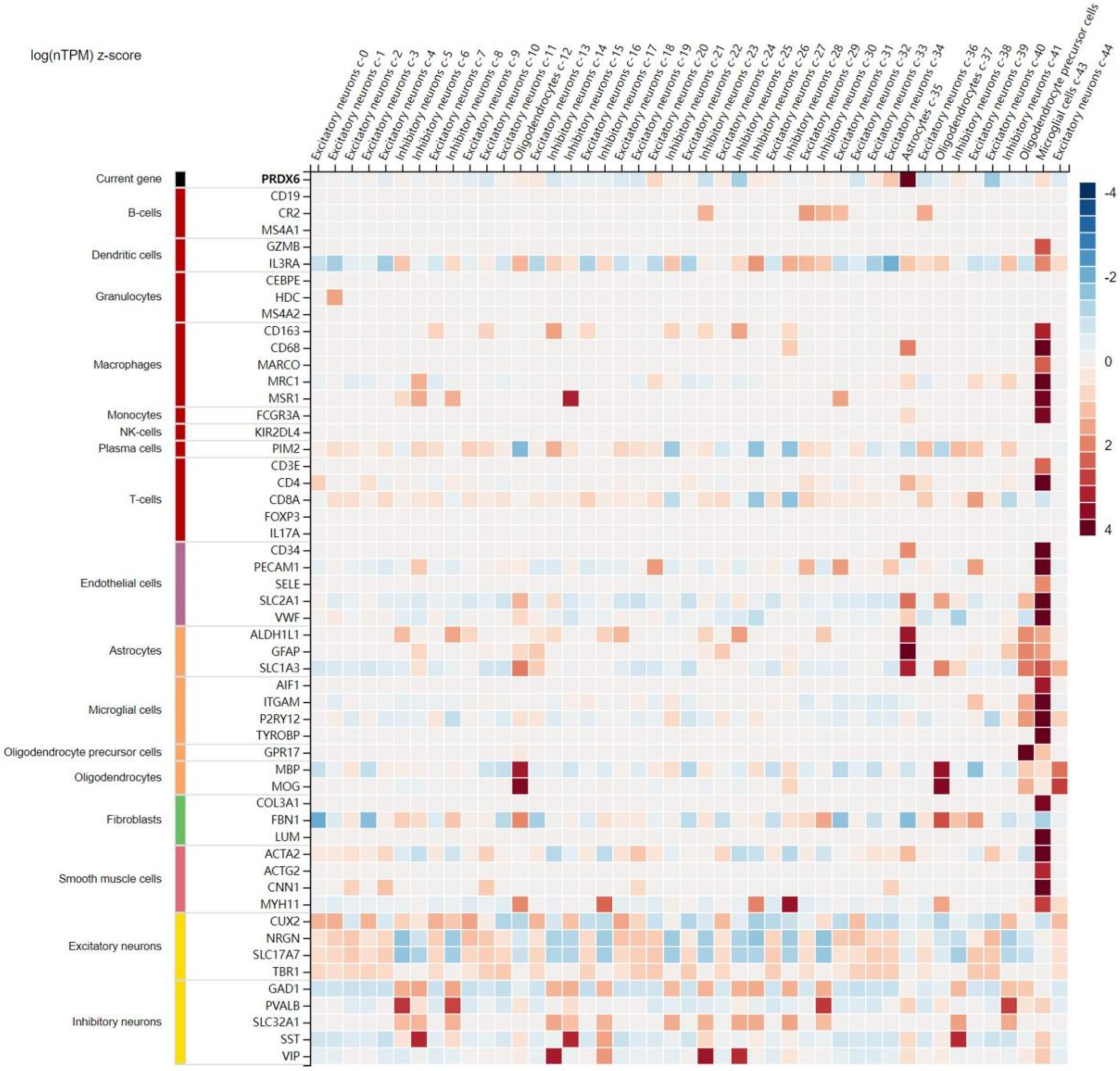

Supplementary Figure 1. The heatmap shows expression of PRDX6 (current gene) and cell type markers in the different single cell type clusters of brain. The panel on the left shows which cell type each marker is associated with, and the color-coding is based on cell types with functional features. Z-scores are the normalization results of mRNA expression levels. (The HPA database)

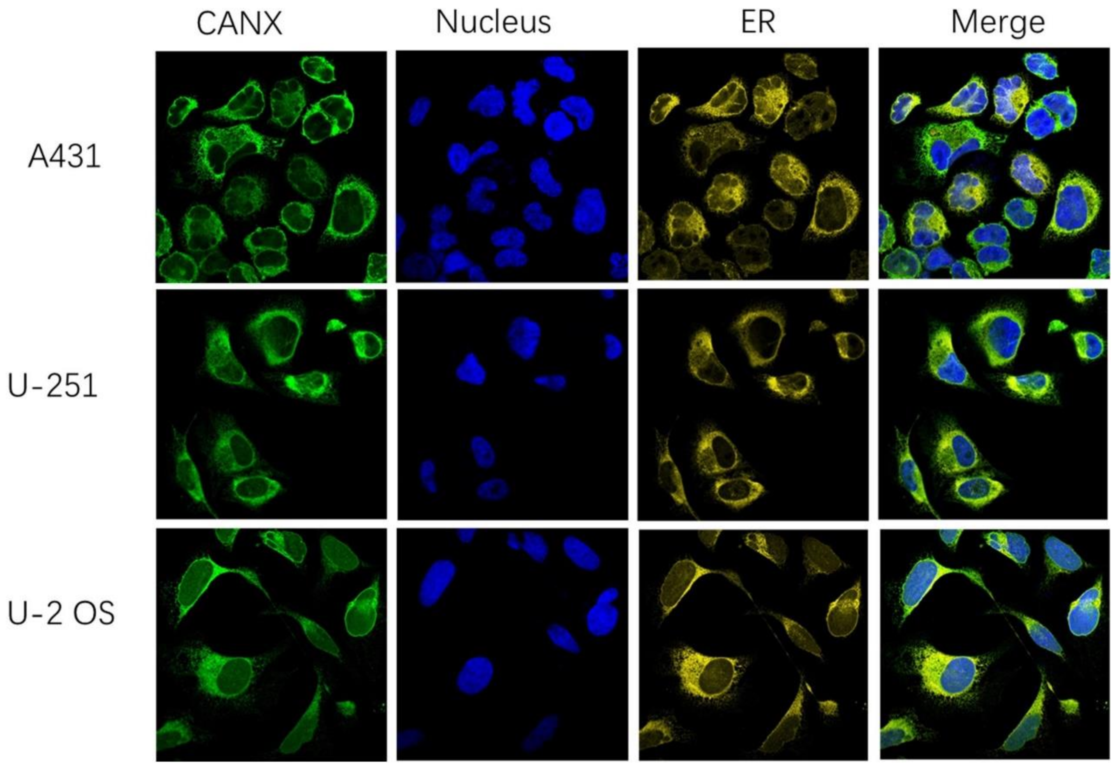

Supplementary Figure 2. The Immunofluorescence results of CANX shows that CANX is detected in the ER. ER, Endoplasmic reticulum. A-431, Epidermoid carcinoma cell line; U-2OS, Osteosarcoma cell line; U-251 MG, Glioblastoma cell line. (The HPA database)
